# Impact of *Ecklonia maxima* Seaweed Extract and Mo Foliar Treatments on Biofortification, Spinach Yield, Quality and NUE

**DOI:** 10.3390/plants10061139

**Published:** 2021-06-03

**Authors:** Salvatore La Bella, Beppe Benedetto Consentino, Youssef Rouphael, Georgia Ntatsi, Claudio De Pasquale, Giovanni Iapichino, Leo Sabatino

**Affiliations:** 1 Dipartimento Scienze Agrarie, Alimentari e Forestali (SAAF), University of Palermo, viale delle Scienze, ed. 5, 90128 Palermo, Italy; salvatore.labella@unipa.it (S.L.B.); claudio.depasquale@unipa.it (C.D.P.); giovanni.iapichino@unipa.it (G.I.); 2 Department of Agricultural Sciences, University of Naples Federico II, 80055 Portici, Italy; youssef.rouphael@unina.it; 3 Laboratory of Vegetable Production, Department of Crop Science, Agricultural University of Athens, 11855 Athens, Greece; ntatsi@aua.gr

**Keywords:** SE-based biostimulant, molybdenum, *Spinacia oleracea* L., plant performance, NUE indices

## Abstract

Seaweed extract (SE) application is a contemporary and sustainable agricultural practice used to improve yield and quality of vegetable crops. Plant biofortification with trace element is recognized as a major tool to prevent mineral malnourishment in humans. Mo deficiency causes numerous dysfunctions, mostly connected to central nervous system and esophageal cancer. The current research was accomplished to appraise the combined effect of *Ecklonia maxima* brown seaweed extract (SE) and Mo dose (0, 0.5, 2, 4 or 8 µmol L^−1^) on yield, biometric traits, minerals, nutritional and functional parameters, as well as nitrogen indices of spinach plants grown in a protected environment (tunnel). Head fresh weight (FW), ascorbic acid, polyphenols, N, P, K, Mg and nitrogen use efficiency (NUE) were positively associated with SE treatment. Moreover, head FW, head height (H), stem diameter (SD), ascorbic acid, polyphenols, carotenoids as well as NUE indices were enhanced by Mo-biofortification. A noticeable improvement in number of leaves (N. leaves), head dry matter (DM) and Mo concentration in leaf tissues was observed when SE application was combined with a Mo dosage of 4 or 8 µmol L^−1^. Overall, our study highlighted that *E. maxima* SE treatment and Mo supply can improve both spinach production and quality via the key enzyme activity involved in the phytochemical homeostasis of SE and the plant nutritional status modification resulting in an enhanced spinach Mo tolerance.

## 1. Introduction

Currently, modern agriculture must face the double task of nourishing the worldwide population and diminishing the ecological impact of horticultural systems [1,2]. One of the most pioneeristic agronomic practices to meet these challenges is the use of plant biostimulants which can elicit growth and development, productivity, abiotic stress tolerance and quality of plants [3,4]. Several authors [5,6,7,8,9,10,11,12] have reported that biostimulants can promote primary and secondary metabolism in vegetables, modulating micro- and macronutrient uptake and assimilation, buildup of phytochemicals and tolerance to abiotic distresses. Among plant biostimulants, seaweed extracts (SEs), especially the brown macro-algae, are often used for their content in signaling molecules such as polysaccharides, betaines, macro- and micronutrients and phytohormones which enhance plant performance [13,14]. The upsurge of crop yields prompted by SE application under optimal or unfavourable cultivation conditions has been linked with a number of physiological and biochemical mechanisms, including the elicitation of enzymes included in carbon and nitrogen metabolic pathways, the Krebs cycle and glycolysis, the stimulation of phytohormones and the boost of mineral uptake and accumulation of treated plants via root morphology alterations [15,16,17].

Along with the persistent concern of maximizing the yield of horticultural crops, there is an urgent request for vegetables of high quality. This is motivated by the increasing attention of consumers to vegetables containing high amounts of nutritional and biofunctional compounds. Furthermore, the enrichment of vegetables with micronutrients (agronomic biofortification) is an essential tool to overcome mineral malnourishment in humans [18,19,20]. Molybdenum (Mo) is a valuable and indispensable trace element to avoid disorders related to the simple deficiency of sulphite oxidase [21,22]. Tsongas et al. [23] communicated an optimal Mo consumption of 120–240 μg per day, dependent on sex, age and income. Generally, Mo can be detected in foods such as legumes, nuts, cereals and cereal derivatives, in form of soluble molybdates [24]. Furthermore, as specified by Pennington et al. [24] and Rose et al. [25], bread and pasta are the principal food providers of dietary Mo ingestion, followed by vegetables.

The benefits of Mo for higher plants are well-known and documented [26,27,28,29]. Plants use Mo in specific enzymes [30] which conduct redox reactions, specifically, in processes comprising nitrogen metabolism [31]. Mo biofortification promotes plant performance in fruiting and green leafy vegetables [32,33]. Moreover, Mo foliar supply in grapevines has proven itself a promising practice to enhance yields [34].

Among green leafy vegetables, spinach (*Spinacia oleracea* L.) is a main crop largely cultivated in the Mediterranean area, both in open-field and protected environments. Its leaves are usually consumed either fresh or after storage using specific preservation techniques. Spinach is one of the less efficient green leafy vegetables in terms of nitrogen uptake and utilization [35], and concomitantly, it requires huge nitrogen supplies to develop and acquire a dark green foliage [36]. This leads spinach to build up large quantities of nitrate in its edible plant part [35].

Considering that: (i) SE application and Mo supply are both simple, effective and practical methods to improve spinach production; (ii) both SE application and Mo-biofortification may improve nitrogen use efficiency in green leafy vegetables like spinach [31,37]; (iii) Mo-biofortification enhances the functional aspect of vegetables [32,33]; (iv) SEs increase plant performance and mineral uptake [13,14]; (v) SEs may improve mineral stress tolerance in plants [3,4], specific investigations are crucial to appraise the combined effect of SE application and Mo-biofortification on spinach plant responses (direct and/or indirect). To the best of our knowledge, no research has been conducted on the combined influence of SE application and Mo biofortification on vegetables. Additionally, the possible influences of SE on productivity and nutritive quality of vegetable crops, including spinach, were mostly studied in soilless systems. Thus, the aim of the current study was to appraise the impact of SE and Mo biofortification foliar treatments on yield and yield-related parameters, minerals, nutritional and functional traits, as well as NUE indices of spinach plants grown in a protected environment.

## 2. Results

The biostimulant action of brown seaweed extract from *Ecklonia maxima* can vary depending on multiple interacting parameters such as genus and species, growth conditions (greenhouse versus open field) and foliar feedings of micronutrients such as Mo. Taking this into account, the overall objective of the current work was a composite examination of yield, quality attributes and NUE in greenhouse spinach through a factorial analysis of the relative effects of seaweed-based biostimulant use and Mo-biofortification.

### 2.1. Production and Biometric Features of Spinach Plants

The combined effect of *Ecklonia maxima* SE and Mo doses on head fresh weight (FW), head height (H) and stem diameter (SD) are shown in Table 1. Statistical analysis for head FW, head H and SD displayed no significant interaction between SE and Mo treatments (Table 1).

When averaged over Mo-biofortification, SE significantly augmented head FW. Not considering the SE application, the highest head FW values were observed in plants supplied with 4 or 8 µmol Mo L^−1^, while the lowest values were documented in control plants (Table 1).

Irrespective of Mo treatments, SE did not significantly affect head H. Conversely, plants treated with 2, 4 or 8 µmol Mo L^−1^ exhibited the highest head H values, whereas control plants and plants supplied with 0.5 µmol Mo L^−1^ had the lowest (Table 1).

Regardless of the Mo supply, SE application significantly decreased SD (Table 1). Specifically, plants supplied with a dosage of 2, 4 or 8 µmol Mo L^−1^ had the highest SD values. Control plants and plants treated with 0.5 µmol Mo L^−1^ had the lowest SD (Table 1).

**Figure 1:**
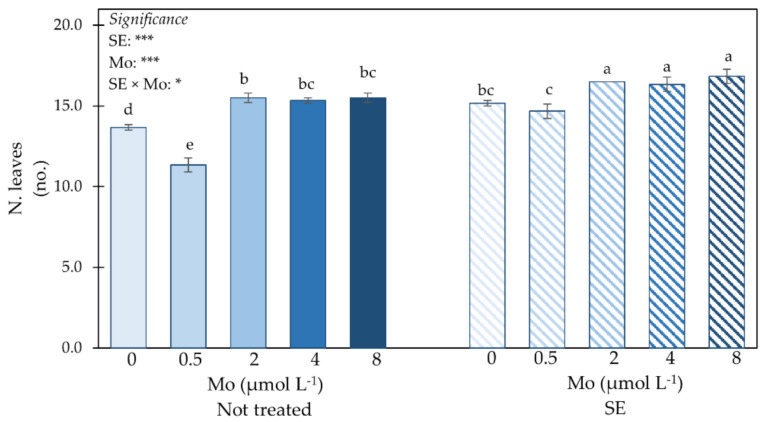
Impact of SE application and Mo-biofortification on number of leaves (N. leaves) of spinach plants cultivated in a protected environment. Values with diverse letters significantly differ at *p* ≤ 0.05. *, *** significant at 0.05 or 0.001, respectively. Bars indicate mean ± standard error of 3 replicates of 5 samples.

The combined effects of *Ecklonia maxima* SE and Mo dose on number of leaves (N. leaves) are presented in Figure 1. ANOVA for N. leaves showed a significant interaction between SE × Mo; plants treated with SE and supplied with 2, 4 or 8 µmol Mo L^−1^ had the highest leaf number, followed by those not treated with SE but fed with 2 µmol Mo L^−1^. The lowest leaf number was recorded in plants not treated with SE and biofortified with 0.5 µmol Mo L^−1^.

### 2.2. Nutritional and Nutraceutical Parameters and Carotenoid Concentration

**Figure 2:**
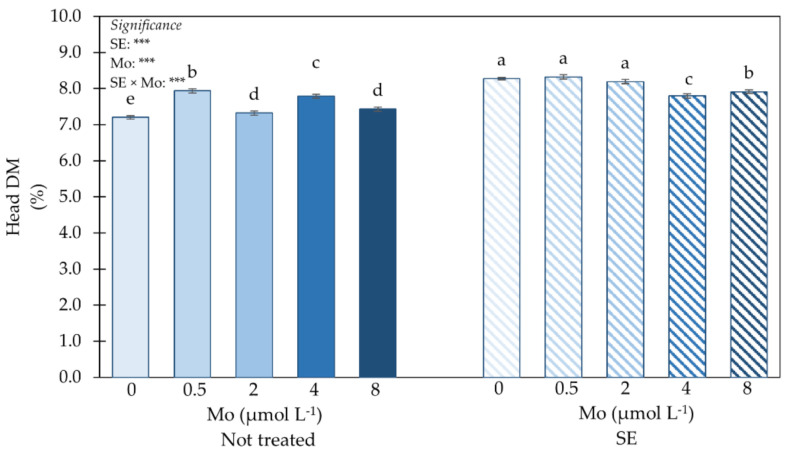
Impact of SE application and Mo-biofortification on head dry matter (head DM) of spinach plants cultivated in a protected environment. Values with diverse letters significantly differ at *p* ≤ 0.05. *** significant at 0.001. Bars indicate mean ± standard error of 3 replicates of 5 samples.

The effect of *Ecklonia maxima* SE and Mo dose on head dry matter (DM) is presented in Figure 2. Statistical analysis for head DM indicated a significant interaction between SE application and Mo-biofortification.

Plants treated with SE and supplied with Mo at 0, 0.5 or 2 µmol L^−1^ had the highest head DM, followed by those untreated with SE and supplied with 0.5 µmol Mo L^−1^ and by those treated with SE with the highest Mo dosage (Figure 2). Untreated plants exhibited the lowest head DM (0 mL SE L^−1^ × 0 Mo µmol L^−1^).

The current study also investigated the impact of *Ecklonia maxima* SE and Mo treatments on color parameters, soluble solids content (SSC), ascorbic acid, polyphenols and carotenoids. Statistics on these parameters indicated no significant interaction between SE × Mo. Treatments had no significant effect on either CIELab parameters or on SSC (Table 2).

Regardless of Mo supply, SE significantly increased ascorbic acid concentration (Table 2). Irrespective of SE application, ANOVA analysis found a positive relation between Mo dosage and ascorbic acid concentration, up to 8 µmol Mo L^−1^ (Table 2). Data on polyphenols supported the trend recognized for ascorbic acid (Table 2). Irrespective of the Mo supply, SE application did not influence carotenoid concentration in leaf tissues. Conversely, ignoring the SE treatments, data on carotenoids sustained the trend previously reported for ascorbic acid and polyphenols (Table 2).

### 2.3. Mineral Concentrations in Leaf Tissues

The analysis of mineral concentrations in leaf tissues was performed to evaluate the influence of *Ecklonia maxima* SE and Mo doses on important nutritional values (Table 3). Statistical analysis for N, P, K, Ca and Mg concentrations did not indicate a significant interaction between SE × Mo.

**Figure 3:**
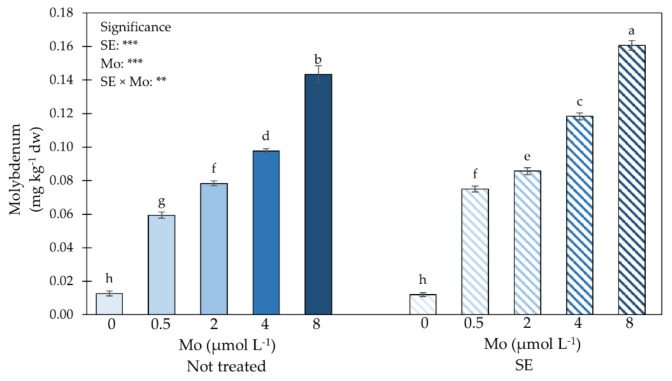
Impact of SE application and Mo-biofortification on molybdenum concentration in leaves of spinach plants cultivated in a protected environment. Values with diverse letters significantly differ at *p* ≤ 0.05. **, *** significant at 0.005 or 0.001, respectively. Bars indicate mean ± standard error of 3 replicates of 5 samples.

Mo leaf enrichment was one of the main purposes of the study. Figure 3 shows that irrespective of the Mo-biofortification, SE treatments significantly augmented N leaf concentration. Control plants had the highest N content followed by plants supplied with 0.5 µmol Mo L^−1^, which in turn had a higher N concentration than those biofortified with Mo at 2 µmol L^−1^. The lowest N leaf concentration was observed in plants treated with the highest Mo dosage (Table 3).

Not considering the Mo treatments, SE application significantly increased P concentration. Regardless of SE treatments, Mo-biofortification did not significantly affect P concentration. Results on K and Mg concentrations support the tendency described for P concentration (Table 3).

SE application and Mo-biofortification did not affect Ca concentration in leaf tissues (Table 3).

ANOVA and mean separation for leaf Mo concentration displayed a significant interaction SE × Mo (Figure 3).

Plants treated with SE and supplied with the highest dosage of Mo had the highest Mo concentration, followed by those non-treated with SE and biofortified with 8 µmol Mo L^−1^, which in turn displayed a higher value than those treated with SE and supplied with 4 µmol Mo L^−1^. The lowest Mo concentration was detected in Mo-untreated plants (Figure 3).

### 2.4. Nitrogen Indices

Since spinach is one of the less efficient green leafy vegetables in terms of nitrogen uptake and utilization, NUE indices represented in this experiment are valid indicators to evaluate the impact of *Ecklonia maxima* SE and Mo doses. ANOVA for NUE, NUpE and NiPUE did not indicate a significant interaction between SE application and Mo doses (Table 4).

Irrespective of Mo-biofortification, SE application significantly increased NUE. Regardless of SE application, plants biofortified with Mo at 4 or 8 µmol L^−1^ had the highest NUE indices, while control plants and plants treated with 0.5 µmol Mo L^−1^ had the lowest. (Table 4).

Regardless of Mo-biofortification, NUpE index was significantly increased by SE application. Regardless of SE treatment, plants supplied with 2, 4 or 8 µmol Mo L^−1^ had the highest NUpE index, whereas control plants the lowest (Table 4).

Not considering Mo-biofortification, SE application significantly decreased the NiPUE index. Notwithstanding SE application, plants biofortified with 8 µmol Mo L^−1^ had the highest NiPUE index and control plants had the lowest (Table 4).

### 2.5. Heat Map Analysis of All Spinach Plant Features

**Figure 4:**
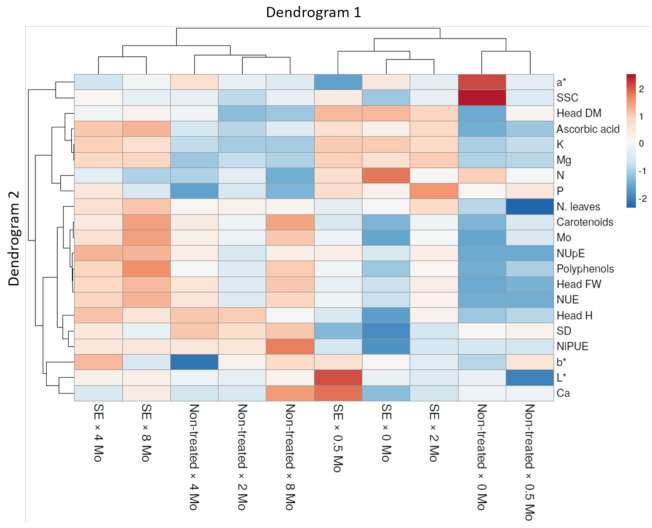
Heat map analysis comprising all spinach plant traits in response to SE application and Mo-biofortification. The heat map picture was made via the https://biit.cs.ut.ee/clustvis/ (accessed on 27 April 2021) online program package.

A grouped data heat map analysis of all agronomic, nutritional, functional and physiological plant traits was carried out to expose a chromatic evaluation of the SE application and Mo-biofortification on spinach plants. In Figure 4, the heat map analysis exposes a couple of dendrograms, one placed on the top and named Dendrogram 1, and the other sited on the left, named Dendrogram 2.

Dendrogram 1 comprises the combinations of SE and Mo treatments, while Dendrogram 2 exhibits all studied features which modified the distribution. Dendrogram 1 displays two principal groups, the one on the left collected the non-treated × 2, 4 and 8 μmol Mo L^−1^ and SE × 4 and 8 μmol Mo L^−1^ combinations. The other on the right side of the cluster includes the non-treated × 0 and 0.5 μmol Mo L^−1^ and SE × 0, 0.5 and 2 μmol Mo L^−1^ combinations (Figure 4).

Specifically, two groups were identified on the left side of Dendrogram 1. The first on the left comprises the SE × 4 μmol Mo L^−1^ and SE × 8 μmol Mo L^−1^ combinations, parted from non-treated × 2, 4 and 8 μmol Mo L^−1^ combinations. These exhibited particularly low values for SSC, head DM, ascorbic acid, K, Mg, N, P, N. leaves, carotenoids, Mo, NUpE, polyphenols, head FW, NUE, b*, L* and Ca. The group on the left encloses SE × 4 Mo and SE × 8 Mo combinations. Inside this group, the SE × 4 μmol Mo L^−1^ combination is evidently separated by higher SSC, K, N, P, head H, SD and b*, whereas the clustering on the right side comprises the non-treated × 2, 4 and 8 μmol Mo L^−1^ combinations. Within this group, the non-treated × 4 μmol Mo L^−1^ combination was parted by higher a*, head DM, K, NUpE and head H and lower Mg, P, N. leaves, polyphenols, NiPUE, b* and Ca. The right side of this cluster encloses non-treated × 2 and 8 μmol Mo L^−1^ combinations. The non-treated × 2 μmol Mo L^−1^ combination was separated by lower SSC, head DM, ascorbic acids, carotenoids, Mo, NUpE, polyphenols, head FW, NUE, SD, NiPUE, b*, L* and Ca (Figure 4).

Looking to the right side of the Dendrogram 1, two clusters were identified. The first cluster on the left encloses SE × 0, 0.5 and 2 μmol Mo L^−1^ combinations separately from the non-treated × 0 and 0.5 μmol Mo L^−1^ combinations, which had higher head DM, ascorbic acid, K, Mg, N, P, N. leaves, carotenoids, Mo, NUpE, polyphenols, head FW, NUE, b*, L* and Ca, but lower a*, SSC, head H, SD and NiPUE. The cluster on the left includes the SE × 0.5 μmol Mo L^−1^ combination; in this group the SE × 0.5 μmol Mo L^−1^ combination is markedly parted by higher SSC, head DM, ascorbic acid, K, Mg, carotenoids, Mo, NUpE, polyphenols, b*, L* and Ca. The right side of this cluster comprises SE × 0 and 2 μmol Mo L^−1^ combinations; the SE × 0 μmol Mo L^−1^ combination was separated by lower SSC, ascorbic acid, Mg, P, N. leaves, carotenoids, Mo, NUpE, polyphenols, head FW, NUE, head H, SD, NiPUE and Ca (Figure 4).

The group on the right side encloses the non-treated × 0 and 0.5 μmol Mo L^−1^ combination. Within this cluster, the non-treated × 0 μmol Mo L^−1^ combination is divided by lower head DM, ascorbic acid, P, carotenoids, Mo, polyphenols, head H, SD and b*, and higher a*, SSC, N, N. leaves and L*. Intriguingly, the groups in Dendrogram 2 manifestly highlight the diverse influence of SE application and Mo-biofortification (Figure 4).

## 3. Discussion

Nowadays, SEs have a reputation as plant biostimulants due to their ability to improve performance, mineral uptake and mineral stress tolerance in vegetable crops [3,4,13,14,17]. Concurrently, plant fertilization management, such as counting trace elements like molybdenum, is an essential cultivation step to the enhance functional aspect (direct advantage) as well as yield and quality (indirect advantages) [32,33]. Furthermore, as reported by several authors [31,37], both SE application and Mo-biofortification may improve nitrogen use efficiency in green leafy vegetables, such as spinach. Thus, in the present study the impact of *Ecklonia maxima* SE and Mo-biofortification foliar treatments, alone or combined, on yield and yield-related parameters, minerals, nutritional and functional traits, as well as NUE indices of spinach was evaluated.

Our results on yield showed that SE enhanced plant productivity. These findings are supported by Rouphael et al. [37] who, by studying the impact of plant and seaweed extracts on spinach cultivated in a protected environment, found that *E. maxima* SE significantly improved yield by 53%. Analogous results were also observed by Di Mola et al. [38], who investigated baby lettuce grown under diverse nitrogen regimes. The higher yield could be related to the SE polysaccharide content. Indeed, sugars are recognized to increase plant productivity by eliciting endogenous hormone homeostasis [39]. We found that the highest Mo supply improved yield and yield-related traits. This is in accordance with the reports of Moncada et al. [32], Biacs et al. [40] and Vieira et al. [41] on leafy vegetables (lettuce, escarole and curly endive, respectively), carrot and common bean.

Our study revealed that SE enhanced head DM percentage. These findings, although in contrast with those of Colla et al. [1] on tomato, are in harmony with those indicated by Rouphael et al. [37] on spinach. These dissimilar results could be related to the different plant organs (leaves *vs* fruits) and species tested. Our results are in accordance with those reported by Boertje [42], who found that Mo-shortage reduces dry matter in lettuce. Plants exposed to SE application had a head DM percentage which did not differ from that of plants treated with 0, 0.5 or 2 µmol Mo L^−1^. Thus, it seems that in our study the SE biostimulant effect was much higher than that of Mo-biofortification and, consequently, SE application produced a buffer effect towards Mo supply. Furthermore, our study underlined that SE-treated plants supplied with the highest Mo dosage (8 µmol L^−1^) displayed a higher head DM percentage than SE-untreated plants. Consequently, we may speculate that SE application enhanced spinach Mo tolerance.

Leaf colour parameters were not influenced by SE application. Our results are sustained by those of Rouphael et al. [37] who found no significant effect of *E. maxima* SE on CIELab parameters in spinach leaves. However, our findings are partially consistent with those reported on baby lettuce plants by Di Mola et al. [38] who found that L* and b* coordinates increased with SE application. Thus, we may hypothesize that the effect of SE treatment on leaf colour is a species-related feature. Our results also revealed that Mo-biofortification did not significantly influence a*, b* and L* color coordinates. These results diverge with those by Moncada et al. [32] on lettuce, escarole and curly endive.

Both SE application and Mo-biofortification did not affect SSC content. These results support those of Colla et al. [1], who detected no significant effect of SE application on total soluble solids content in tomato, but differ with those of Moncada et al. [32], who found that Mo supply increased total soluble solids content. These dissimilarities could be related to the different effects of Mo availability on the metabolism of diverse species. This hypothesis is supported by Kaiser et al. [31] who specified that, since Mo is an element included in several enzymatic mechanisms, it can be difficult to identify a definite plant reaction to its deficiency.

Our results revealed that SE application improved ascorbic acid and polyphenol content in spinach leaves. These outcomes support the findings of Rouphael et al. [37] on spinach and those of Abbas et al. [43] on onion. The elicitation of the secondary metabolism, resulting in an augment of bioactive molecules (i.e., total phenols and ascorbic acid), could be related to the key enzyme activity (chalcone isomerase) in phytochemical homeostasis [44,45]. Furthermore, the secondary metabolism stimulation could be linked to the modification of the plant nutritional status (indirect effect) [7]. Likewise, as reported by Fan et al. [46], SE of *A. nodosum* application improved flavonoid concentration in spinach-treated plants compared to the control. Our results also displayed that ascorbic acid and polyphenol concentrations increased as Mo dosage increased. Similar findings are reported on lettuce, cauliflower, tomato and potatoes [33,42,47,48]. Ascorbic acid has a significant role in chloroplast protection. According to Valenciano et al. [49], the reduction in ascorbic acid concentration in Mo-deficient plants might be connected to the chloroplast inefficiency that occurs in plants with Mo deficiency.

Our results displayed that SE application did not significantly affect carotenoid concentration. This is in contrast with the outcome of Di Mola et al. [38], who reported a beneficial effect of the SE-based biostimulant on baby lettuce. However, Carillo et al. [50], by studying the influence of protein hydrolysate (PH) and different nitrogen levels in spinach, found that carotenoid content was negatively affected by PH application. Thus, we may assume that plant- or seaweed-based biostimulants could affect carotenoid content differently based on genotype. Furthermore, our study highlighted that a higher Mo concentration in the nutrient solution increased carotenoid concentration. Since Mo supply improves plant nitrogen metabolism [51], and considering that nitrogen fertilization enhances carotenoid concentration in plants [38,52], we hypothesized that in our study Mo supply indirectly increases carotenoid concentration.

Regarding the mineral profile of spinach, we found that SE application enhanced N, P, K and Mg concentration in leaves, whereas it did not affect Ca concentration. Our outcomes are partially consistent with those by Rouphael et al. [37], who found that SE application causes an increase in protein, K and Mg, without affecting Ca and P concentrations. Our outcomes were also in line with those of Di Mola et al. [38], who reported that, notwithstanding the nitrogen rates, SE improved nitrate concentration in baby lettuce leaves. These results could be attributed to the fact that SE changes the root architecture, resulting in improved plant nutrient uptake [17]. Furthermore, as reported by Luthje and Bottger [53], SE contains a component called kahydrin, a derivative of vitamin K1, which alters the plasma membrane proton pumps and stimulates the secretion of H^+^ ions into the apoplast, leading to rhizosphere acidification. This condition modifies the soil redox state and metal ion solubility, increasing their availability to the plant [54,55]. Our findings reveal that Mo supply did not significantly influence P, K, Ca and Mg concentrations in spinach plants, but significantly decreased N concentration in leaf tissues. Our outcomes are consistent with those of Moncada et al. [32], who found a reduced nitrate content in Mo-biofortified green leafy vegetables. Our findings are also corroborated by those of Zhen et al. [56], Cantliffe et al. [57] and Cox [58]. Moreover, molybdenum cofactors (Moco) partake in the active site of nitrate reductase, which modulates nitrate uptake and may improve nitrogen use efficiency [30].

A significant influence of the interaction SE application x Mo supply on leaves Mo concentration was found in this study. In particular, plants supplied with the highest Mo dosage and treated with SE provided the highest Mo concentration in leaves. Conversely, control plants (0 µmol Mo L^−1^) treated or not with SE showed the lowest Mo content. Our findings are corroborated by those of Colla et al. [1], who stated that plant-based biostimulants improve plant macro- and micronutrient absorption and accumulation. Our results are also in line with those of Moncada et al. [32], who found a positive correlation between Mo concentration in the nutrient solution and Mo leaves tissue content. However, in our study when plants were not biofortified with Mo, SE application did not enhance Mo concentration. Thus, we assume that to optimize Mo content in spinach plants, the combined use of Mo-biofortification and SE application is crucial.

Our results indicate that both SE application and Mo supply enhanced nitrogen use efficiency indices. These outcomes could be linked to the fact that SE modifies the roots architecture, enhancing the efficiency of plant mineral absorption [17]. Furthermore, it is known that Mo supply, via the Moco participation in nitrate reductase, modulates nitrogen uptake [49] and consequently improves nitrogen use efficiency.

## 4. Materials and Methods

### 4.1. Plant Genetic Resource and Research Location

**Figure 5:**
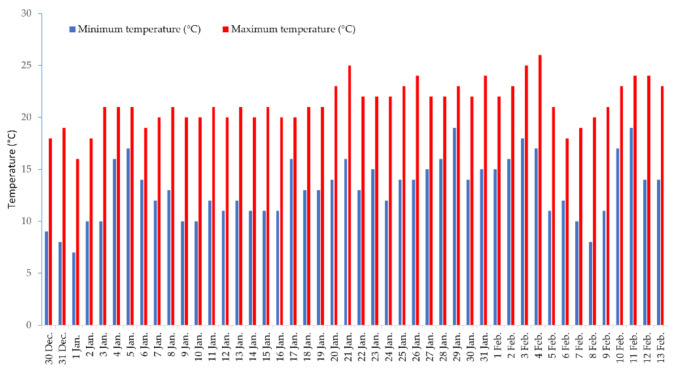
Maximum and minimum temperature registered daily from 30 December to 13 February at the experimental station (latitude 38.12° N, longitude 13.36° E, altitude 65 m).

The research was conducted in Palermo (Italy), at an experimental field of the Department of Agricultural, Food and Forestry Sciences of the University of Palermo (latitude 38.12° N, longitude 13.36° E, altitude 65 m). The study was accomplished in a tunnel covered with a transparent polyethylene film (0.05 mm in thickness) and provided with a micro-flow irrigation system with dripping wings placed on the ground. Daily maximum and minimum temperatures during the growing cycle were monitored and collected via a data logger located inside the tunnel, 1.5 m from the ground (Figure 5).

On 30 December 2020, plug plants of spinach (*Spinacia oleracea* L.) Spargo F_1_ (Fratelli Ingegnoli, Milan, Italy), were transplanted at the 4–5 true leaves stage with a plant density of 25 plants per m^−2^ (20 cm between and inter rows). Through the whole cultivation cycle, spinach plant demands were ensured following all conventional cultivation practices [59]. All plants were harvested 45 days after transplant.

### 4.2. Experimental Set-Up and Design

The seaweed treatment was accomplished using a liquid extract of *Ecklonia maxima* (Kelpstar^®^, Mugavero fertilizers, Palermo, Italy) produced through a cold micronization process, which does not use heat or chemicals. It contains organic nitrogen (1%), organic carbon (10%), phytohormones; mainly auxin (11 mg L^−1^) and cytokinin (0.03 mg L^−1^) and organic substances with nominal molecular weights < 50 kDa (30%). The pH of Kelpstar^®^ is 6.0. Foliar spray SE treatments started seven days after transplant and were distributed weekly. Mo was distributed via foliar spray in form of sodium molybdate (Na_2_MoO_4_). Mo treatments started ten days after transplant and were accomplished every ten days. For every foliar spray application, the volume used was 1.0 L m^−2^. Two doses of SE [0 (control treatment) or 3 mL L^−1^ (recommended dosage)] were combined with five molybdenum (Mo) doses [0.0 (control), 0.5, 2.0, 4.0 or 8.0 µmol L^−1^]. The treatments were replicated 3 times (15 plants each one) and organized in a randomized complete block design, rendering 30 experimental units (2 SE × 5 Mo × 3 replicates).

### 4.3. Production, Nutritional, Nutraceutical Implications and Carotenoid Concentration of Spinach Plants

Five samples, randomly selected from each replicate, were used for carrying out all agronomic and qualitative traits of Spargo F_1_ spinach. Immediately after harvesting, all plants were washed with deionized water to remove residual components of the treatments.

Values of head fresh weight (head FW), head height (head H), stem diameter (SD) and number of leaves (N. leaves) are considered the main yield and yield-related traits in spinach, thus, they were collected instantaneously after the harvest. Since spinach is a green leafy vegetable, we did not consider it relevant to collect data on days to flowering. Our study focused on the assessment of the most critical nutritional and functional features of spinach. To appraise soluble solid content (SSC), 100 g of leaf sample was squeezed and filtered, then the measurement was carried out via a digital refractometer (MTD-045 nD, Three-In-One Enterprises Co. Ltd. New Taipei, Taiwan). The SSC value is reported as Brix°.

After plant harvest, CIELab color parameters (a*, b* and L*) were measured via a colorimeter (Chroma-meter CR-400, Minolta corporation Ltd., Osaka, Japan) on five intact leaves casually chosen from each replicate.

To evaluate percentage of dry matter (percentage DM), leaf samples were dried in an oven at 105 °C up to constant mass. The concentration of ascorbic acid in leaves was revealed using a reflectometer (Merck RQflex10 Reflectoquant^®^, Sigma-Aldrich Saint Louis, MO, USA) and reflectoquant ascorbic acid test strips (Merck, Darmstadt, Germany). Ascorbic acid value is expressed as mg 100 g^−1^ fresh weight.

Polyphenol concentration was assessed by the Folin-Ciocalteau method [60]. In brief, leaf tissue samples were mixed with Folin-Ciocalteau reagent, distilled water and sodium carbonate, then the mixture was kept in room temperature for 30 min. After that, the absorbance of the solution was evaluated at 750 nm via a spectrophotometer. The polyphenol concentration is expressed as gallic acid equivalents (GAE) 100 g^−1^ dry weight. Plant carotenoid concentration was assessed following the method reported by Costache et al. [61]. Briefly, a sample of 1 g was mixed with methanol, then the measurement was conducted via a spectrophotometer. The carotenoid concentration value is expressed as µg g^−1^ dry weight.

### 4.4. Mineral Profile

For calcium (Ca), magnesium (Mg) and potassium (K) concentrations, the method described by Morand and Gullo [62] was applied. Phosphorous (P) concentration was determined using the Fogg and Wilkinson [63] method. Nitrogen (N) concentration in leaf tissues was measured following the Kjeldahl method. Molybdenum (Mo) concentration was evaluated as reported by Sabatino et al. [33] via inductively coupled plasma-mass spectrometry (ICP-MS) (Plasma Quant MS Elite, Jena, Germany). The concentrations of N, P, K, Ca and Mg are expressed as g kg^−1^ dry weight, while the Mo concentration is shown as mg kg^−1^ dry weight.

### 4.5. Calculation of Nitrogen Indices

Nitrogen use efficiency (NUE), nitrogen uptake efficiency (NUpE) and nitrogen physiological use efficiency (NiPUE) were determined as follows: NUE = yield (t)/N application rate (kg); NUpE = plant N content (kg)/N application (kg); NiPUE = yield (t)/plant nitrogen content (kg).

### 4.6. Statistics and Heat Map

The SPSS software v.20 (StatSoft, Inc., Chicago, USA) package was used to analyze all datasets through a two-way Analysis Of Variance (ANOVA). Tukey Honestly Significant Difference (HSD) test (*p* < 0.05) was adopted for multiple comparisons of means. Data expressed as percentages were converted via arcsine transformation prior to ANOVA analysis (Ø = arcsine(p/100)1/2).

A heat map revealing the agronomical, qualitative and NUE responses of spinach to SE applications and Mo supply dosages was also created.

## 5. Conclusions

The continuous need to maximize yield and functional action of green leafy vegetables imposes a challenge for growers, extension specialists and researchers in their search for eco-friendly tools to combine high production with premium quality. In the present study, *E. maxima* SE significantly enhanced yield parameters, mineral profile, nutritional and functional features, and NUE indices. Concurrently, Mo supply substantially increased productivity, NUE indices, nutritional and bioactive features such as ascorbic acid, polyphenols, Mo leaf concentration and carotenoids, while it decreased N content in leaf tissues. Despite the remarkable current literature on the use of SE biostimulants alone as well as that of biofortification with trace elements, information regarding the combined effect of both agronomic practices is limited. In view of the above considerations, our results showed that combining SE with 2 μmol Mo L^−1^ notably ameliorated leaf number and head DM, whereas, combining SE with 8 μmol Mo L^−1^ significantly enhanced leaf Mo concentration. Overall, our novel outcomes recommend that a mutual application of SE and Mo supply at 4 or 8 μmol L^−1^ may efficiently increase crop performance and the nutritional and functional quality of spinach.

## Figures and Tables

**Table 1:** Effect of SE application and Mo-biofortification on head fresh weight (head FW), head height (head H) and stem diameter (SD) of spinach plants cultivated in a protected environment.

Treatments	Head FW (g)	Head H (cm)	SD (mm)	
*SE (ml L^−1^)*				
0	39.73 b	15.64 a	9.83	a
3	45.44 a	15.52 a	8.23	b
*Mo (µmol L^−1^)*				
0	32.09 c	13.30 c	8.00	c
0.5	35.07 bc	14.35 bc	8.41	bc
2	42.32 b	16.68 ab	9.17	ab
4	49.67 a	17.44 a	10.00	a
8	53.49 a	16.11 ab	9.58	a
*Significance*				
SE	***	NS	***	
Mo	***	***	***	
SE × Mo	NS	NS	NS	

Values in a column with diverse letters significantly differ at *p* ≤ 0.05. NS, *** not significant or significant at 0.001, respectively. Each value is the mean of 3 replicates of 5 samples each.

**Table 2:** Effect of SE application and Mo-biofortification on CIELab parameters (a*, b* and L*), soluble solid content (SSC), ascorbic acid, polyphenols and carotenoids of spinach plants cultivated in a protected environment.

Treatments	a*	b*	L*	SSC (Brix°)	Ascorbic Acid (mg 100 g^−1^ pf)	Polyphenols (GAE 100g^−1^ ps)	Carotenoids (µg g^−1^ ps)
*SE (ml L^−1^)*							
0	−15.73a	16.79 a	34.31 a	3.04 a	87.87 b	29.27 b	5.48 a
3	−17.22 a	17.11 a	36.32 a	3.02 a	137.36 a	31.63 a	5.47 a
*Mo (µmol L^−1^)*							
0	−14.92 a	16.72 a	34.93 a	3.09 a	97.05 e	25.98 e	5.19 e
0.5	−16.87 a	17.38 a	35.52 a	3.03 a	107.85 d	28.62 d	5.36 d
2	−16.27 a	16.91 a	34.61 a	2.99 a	113.03 c	30.05 c	5.46 c
4	−16.06 a	16.67 a	35.56 a	3.03 a	119.90 b	32.31 b	5.58 b
8	−16.25 a	17.06 a	35.97 a	3.02 a	125.23 a	35.28 a	5.78 a
*Significance*							
SE	NS	NS	NS	NS	***	***	NS
Mo	NS	NS	NS	NS	***	***	***
SE × Mo	NS	NS	NS	NS	NS	NS	NS

Values in a column with diverse letters significantly differ at *p* ≤ 0.05. NS, *** not significant or significant at 0.001, respectively. Each value is the mean of 3 replicates of 5 samples each.

**Table 3:** Impact of SE application and Mo-biofortification on mineral profile (N, P, K, Ca and Mg) of spinach plants cultivated in a protected environment.

Treatments	N (g kg^−1^ dw)	P (g kg^−1^ dw)	K (g kg^−1^ dw)	Ca (g kg^−1^ dw)	Mg (g kg^−1^ dw)
*SE (ml L^−1^)*					
0	5.35 b	3.61 b	70.38 b	12.79 a	7.67 b
3	5.50 a	3.70 a	82.51 a	12.78 a	8.66 a
*Mo (µmol L^−1^)*					
0	5.78 a	3.67 a	76.62 a	12.67 a	8.13 a
0.5	5.54 b	3.71 a	77.25 a	12.95 a	8.21 a
2	5.40 c	3.69 a	75.83 a	12.68 a	8.25 a
4	5.27 d	3.61 a	77.05 a	12.68 a	8.14 a
8	5.14 e	3.58 a	75.48 a	12.97 a	8.20 a
*Significance*					
SE	***	*	*	NS	***
Mo	***	NS	NS	NS	NS
SE × Mo	NS	NS	NS	NS	NS

Values in a column with diverse letters significantly differ at *p* ≤ 0.05. NS, *, *** not significant, significant at 0.05 or 0.001, respectively. Each value is the mean of 3 replicates of 5 samples each.

**Table 4:** Impact of SE application and Mo-biofortification on nitrogen use efficiency (NUE), nitrogen uptake efficiency (NUpE) and nitrogen physiological use efficiency (NiPUE) of spinach plants cultivated in a protected environment.

Treatments	NUE (t kg^−1^)	NUpE (kg kg^−1^)	NiPUE (t kg^−1^)
*SE (ml L^−1^)*			
0	0.124 b	0.050 b	0.187 a
3	0.142 a	0.063 a	0.182 b
*Mo (µmol L^−1^)*			
0	0.100 c	0.045 c	0.173 e
0.5	0.109 c	0.049 bc	0.180 d
2	0.132 b	0.056 ab	0.184 c
4	0.156 a	0.064 a	0.189 b
8	0.167 a	0.066 a	0.194 a
*Significance*			
SE	***	***	***
Mo	***	***	***
SE × Mo	NS	NS	NS

Values in a column with diverse letters significantly differ at *p* ≤ 0.05. NS, *** not significant or significant at 0.001, respectively. Each value is the mean of 3 replicates of 5 samples each.

## Data Availability

Not applicable.

## Abbreviations

SE	Seaweed extract
Mo	Molybdenum
N	Nitrogen
P	Phosphorus
K	Potassium
Ca	Calcium
Mg	Magnesium
ANOVA	Analysis of Variance
NUE	Nitrogen Use Efficiency
NUpE	Nitrogen Uptake Efficiency
NiPUE	Nitrogen Physiological Use Efficiency
Head FW	Head Fresh Weight
Head H	Head Height
SD	Stem Diameter
N. leaves	Number of Leaves
Head DM	Head Dry Matter
SSC	Soluble Solid Content
